# Infective endocarditis caused by Staphylococcus aureus in a patient with atopic dermatitis: a case report

**DOI:** 10.1186/1752-1947-2-143

**Published:** 2008-05-04

**Authors:** Gadha Mohiyiddeen, Ian Brett, Edward Jude

**Affiliations:** 1 Department of Medicine and Department of Radiology, Tameside General Hospital, Ashton-Under-Lyne, Lancashire OL6 9RW, UK

## Abstract

**Introduction-:**

Atopic dermatitis (AD) is a common condition in the United Kingdom with the prevalence varying from 21% in infants aged 0–6 months to 6.4% at the age of 16 years. Patients with AD experience high rates of colonization of their skin surfaces by Staphylococcus aureus (S. aureus). In severe AD there is a potential risk of staphylococcal bacteremia and invasive infection such as acute endocarditis.

**Case presentation-:**

We report a case of acute endocarditis with mitral valve destruction caused by S. aureus in a 30-year-old man with severe AD. The patient received intensive inpatient treatment with antibiotics and underwent successful mitral valve replacement and skin treatment for AD.

**Conclusion-:**

Patients with severe AD are at higher risk of staphylococcal bacteremia and endocarditis. Staphylococcal endocarditis has to be considered in the differential diagnosis of febrile illness in patients with uncontrolled atopic dermatitis.

## Introduction

AD is a common skin disorder where there is excessive formation of IgE antibodies to inhaled, injected or ingested allergen. The cardinal feature of AD is itch, and scratching may account for most of the signs. Colonization of S. aureus is commonly observed in skin lesions of atopic dermatitis patients, and scratching of the pruritic lesions may lead to reiterative bacteremia and endocarditis. S. aureus typically causes acute endocarditis with damage to cardiac valves, embolisation of vegetation to extracardiac sites and progresses to death within weeks if left untreated. There has been rising awareness in the medical literature about the potential risk of staphylococcal endocarditis in young patients with AD. Although the incidence of endocarditis in atopic dermatitis is rare, this needs to be recognized as one possible complication in this common skin disorder.

## Case presentation

A 30-year-old man with history of AD presented to the accident and emergency department of our hospital with fever and generalized skin rash. He complained of malaise and poor appetite for a week. He had a brief period of confusion on the day prior to admission. He also suffered from bronchial asthma that was well controlled with bronchodilator inhalers. There was no previous history of heart disease or rheumatic fever. He worked as an engineer in a plastic factory. He denied smoking or intravenous drug use. He had extremely dry skin with lichenifications affecting almost all areas of his body but worse on the elbows and knees for which he was applying moisturizers and 1% hydrocortisone cream. The exacerbations of eczema were treated with 2.5% hydrocortisone cream and varying dose of prednisolone tablets.

**Figure 1:**
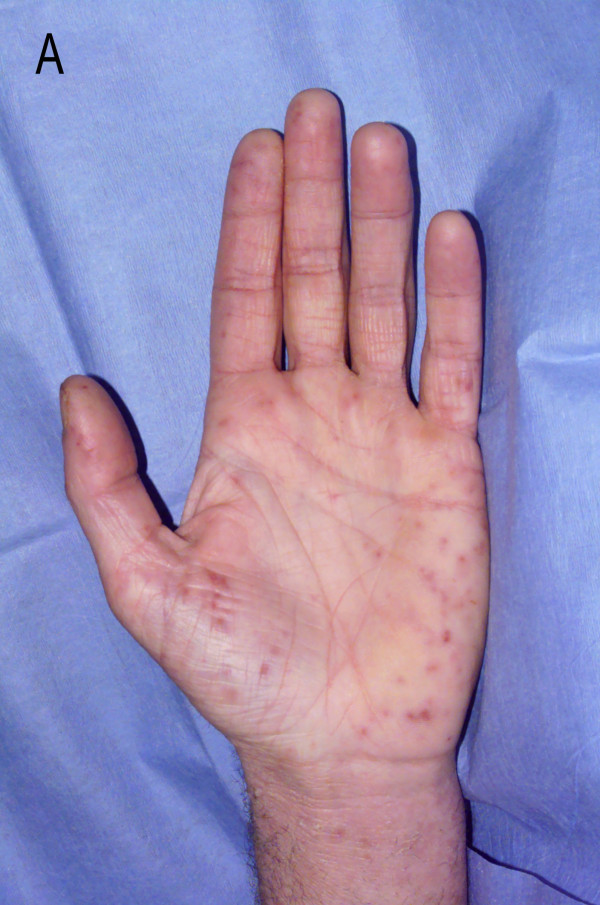
Erythematous macular non-blanching skin rash.

On examination, the patient was drowsy; temperature was 38°C, pulse rate 110/min and blood pressure 144/88 mm Hg. He had a generalized erythematous macular non-blanching skin rash (Figure 1) and small purpuric haemorrhages over the palate. Systemic examination revealed a pansystolic murmur in the mitral area, splenomegaly and weakness of the right leg (power 3/5). Both plantar reflexes were extensor and sustained ankle clonus was present. There were no signs of meningeal irritation, cerebellar dysfunction or sensory deficit and fundoscopic examination was normal.

Laboratory investigations revealed haemoglobin 15.7 g/dl, WBC 14.5 × 10^9^/l, platelets 50 × 10^9^/l, ESR 19 mm/hour, C-reactive protein 292.9 mg/l, serum sodium 128 mmol/l, serum potassium 3.3 mmol/l, bicarbonate 27 mmol/l, blood urea 7.5 mmol/l and serum creatinine 207 μmol/l (patient's baseline – 118 μmol/l). Urine dipstick was positive for blood (++) and protein (+). Chest X-ray was normal. 12 lead ECG showed sinus tachycardia with features of left ventricular hypertrophy. Transthoracic echocardiogram showed mitral valve vegetation with severe mitral regurgitation and normal ejection fraction. CT scan of the brain was normal but MRI identified multiple areas of white matter abnormality suggestive of embolism around the periventricular area. Two sets of blood cultures grew Staphylococcus aureus sensitive to gentamicin and flucloxacillin.

The patient was commenced on intravenous gentamicin and flucloxacillin and later teicoplanin. After 48 hours he became afebrile; the rashes started to fade and his right leg weakness improved. CRP and ESR gradually reduced. Three weeks later, he had a recurrence of fever with elevation of CRP and ESR. Blood culture at that time showed no growth but his central venous line tip grew a coagulase-negative staphylococcus sensitive to rifampicin, vancomycin and netilmycin. His condition improved with removal of the central line and addition of rifampicin. The treatment was continued with teicoplanin for 6 weeks and rifampicin for 4 weeks. He was discharged 5 weeks later on treatment with ramipril and frusemide in view of significant mitral regurgitation.

**Figure 2:**
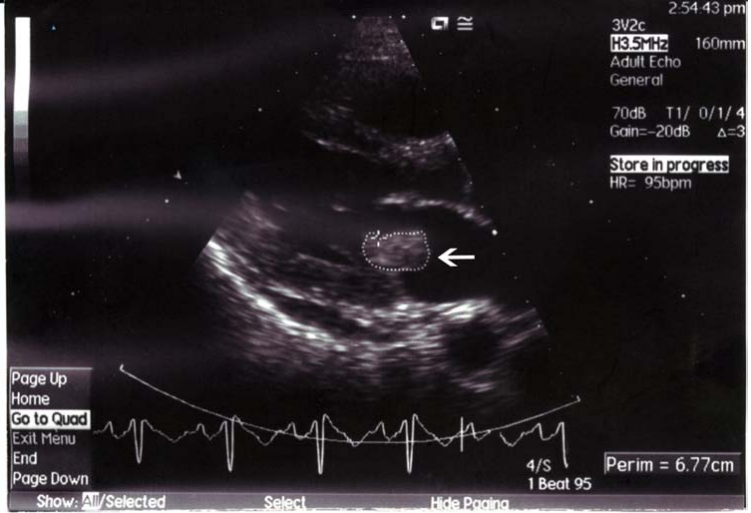
Transoesophageal echocardiogram showing perforation of the posterior mitral cusp with significant mitral regurgitation (spontaneous echo contrast in left atrium).

Three months later, he underwent transoesophageal echocardiogram which showed perforation of the posterior mitral cusp with significant mitral regurgitation (Figure 2). He underwent successful mitral valve replacement 5 months following the presenting episode of infective endocarditis and was commenced on warfarin. Though he had an episode of eczema herpeticum two years following the infective endocarditis, he continues to do well.

## Discussion

This case report lends support to the association of infective endocarditis due to S. aureus and AD. In native valve endocarditis, S. aureus accounts for 30–35% of cases, whereas in a patient with atopic dermatitis, it is found to be exclusively due to S. aureus [1]. This could be the result of frequent staphylococcal bacteremia in patients with AD.

S. aureus is an aggressive pathogen and bacteremia with this organism can infect healthy heart valves. Neurological complications of infective endocarditis, particularly embolic events, tend to be higher with this organism. Preservation of the mitral valve is also rare when infection is caused by S. aureus due to valvular damage and the presence of huge mobile vegetations [2].

A study on the microbial flora of patients with AD revealed carriage rates of S. aureus as 79% in the anterior nares, 76% in the uninvolved normal skin and 93% in the lesions. Thus S. aureus is the predominant organism in the atopic lesions and constituted 91% of the total aerobic bacterial flora. Coagulase-negative staphylococci are the second predominant organisms (9%) isolated from the skin lesions in AD. On normal skin, coagulase-negative staphylococcus is found to be the predominant organism, constituting 63% of the total flora, followed by S aureus, constituting 30% of the bacterial flora [3].

There are several possible causes for the predominant colonization by S. aureus. One study suggested the cause could be due to the lack of the innate immune system of antimicrobial peptides known as cathelicidins (LL-37) and beta-defensins (HBD-2) of the atopic skin. The combination of LL-37 and beta-defensins (HBD-2) is shown to have synergistic antimicrobial activity in the body for effective killing of S. aureus. A deficiency in the expression of antimicrobial peptides in these patients may account for the susceptibility to skin infection with S. aureus [4]. Thus there is a potential risk of staphylococcal bacteremia and acute native valve endocarditis in patients with uncontrolled AD; the latter condition may be a risk factor for the former [2,5]. While the true incidence of systemic staphylococcal infections in AD is currently unknown, it is conceivable that it may be more common than previously thought.

## Conclusion

High rate of cutaneous colonization by S. aureus in atopic dermatitis lesions represents an important source of bacteremia and there is a possibility of bacteremia progressing to invasive bacterial infection such as endocarditis. Staphylococcal bacteremia has to be considered in the differential diagnosis of fever in patients with severe AD [6].

## Competing interests

The authors declare that they have no competing interests.

## Authors' contributions

GM contributed to acquisition of data and drafting the manuscript. IB revised critically for important intellectual content. EJ gave final approval of the version to be published. All authors read and approved the final manuscript.

## Consent

The authors declare that informed written consent was obtained from the patient for the publication of this manuscript and the accompanying figures.
